# Impact of airborne algicidal bacteria on marine phytoplankton blooms

**DOI:** 10.1093/ismejo/wrae016

**Published:** 2024-03-05

**Authors:** Naama Lang-Yona, J Michel Flores, Tal Sharon Nir-Zadock, Inbal Nussbaum, Ilan Koren, Assaf Vardi

**Affiliations:** Department of Plant and Environmental Science, Weizmann Institute of Science, Rehovot 7610001, Israel; Technion - Israel Institute of Technology, Environmental, Water and Agricultural Engineering, Haifa 3200003, Israel; Department of Earth and Planetary Sciences, Weizmann Institute of Science, Rehovot 7610001, Israel; Department of Plant and Environmental Science, Weizmann Institute of Science, Rehovot 7610001, Israel; Department of Plant and Environmental Science, Weizmann Institute of Science, Rehovot 7610001, Israel; Department of Earth and Planetary Sciences, Weizmann Institute of Science, Rehovot 7610001, Israel; Department of Plant and Environmental Science, Weizmann Institute of Science, Rehovot 7610001, Israel

**Keywords:** marine bioaerosols, ocean–atmosphere interactions, airborne pathogen, oceanic blooms, aerial infection, Gephyrocapsa huxelyi

## Abstract

Ocean microbes are involved in global processes such as nutrient and carbon cycling. Recent studies indicated diverse modes of algal–bacterial interactions, including mutualism and pathogenicity, which have a substantial impact on ecology and oceanic carbon sequestration, and hence, on climate. However, the airborne dispersal and pathogenicity of bacteria in the marine ecosystem remained elusive. Here, we isolated an airborne algicidal bacterium, *Roseovarius nubinhibens*, emitted to the atmosphere as primary marine aerosol (referred also as sea spray aerosols) and collected above a coccolithophore bloom in the North Atlantic Ocean. The aerosolized bacteria retained infective properties and induced lysis of *Gephyrocapsa huxleyi* cultures.This suggests that the transport of marine bacteria through the atmosphere can effectively spread infection agents over vast oceanic regions, highlighting its significance in regulating the cell fate in algal blooms.

## Graphical Abstract

**Graphical Abstract:**
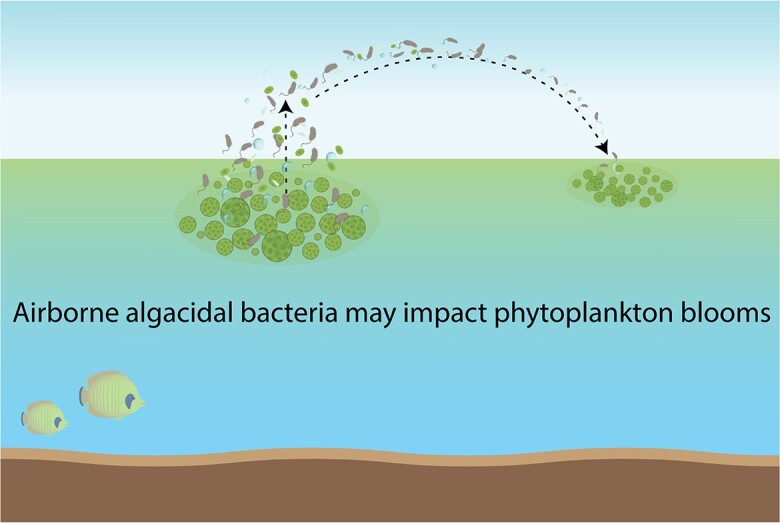

## Introduction

Phytoplankton communities contribute to about half of the estimated global net primary production and are a key component of large biogeochemical cycles in the ocean [1]. *Gephyrocapsa huxleyi* (previously known as *Emiliania huxleyi*) [2] is one such bloom-forming coccolithophore [3]. Massive *G. huxleyi* blooms occur annually and can reach thousands of square kilometres [4], while its cells can be subjected to diverse abiotic (e.g., light regime and nutrient limitation) and biotic interactions with pathogens and grazers [3]. These combined factors regulate the algal cell fate and can eventually lead to bloom demise. The synchronized demise of oceanic *G. huxleyi* blooms, occurring over thousands of kilometres, raises intriguing questions regarding the mortality factors that can regulate cell fate in the bloom and their mode of transmission over large scales. However, the contribution of an aerial infection mechanism to *G. huxleyi* bloom demise is still underexplored. Several dispersal mechanisms were suggested for viral transmission in algal blooms, including emissions of aerosolized viruses [5–7]. The suggested pathogenic vectors were tested under controlled laboratory conditions; however, direct isolations of infective algal pathogens from air samples were never achieved. In this study, we explored the pathogenic potential of airborne bacteria collected above a *G. huxleyi* bloom in the North Atlantic Ocean. We isolated the aerosolized bacterium, *Roseovarius nubinhibens*, and demonstrated that it can remain infective as sea spray aerosol (SSA; aerosols directly emitted from the ocean surface) and keep its pathogenic activity against healthy *G. huxleyi* cultures.

## Materials and methods

### Oceanographic cruise sampling

**Figure 1:**
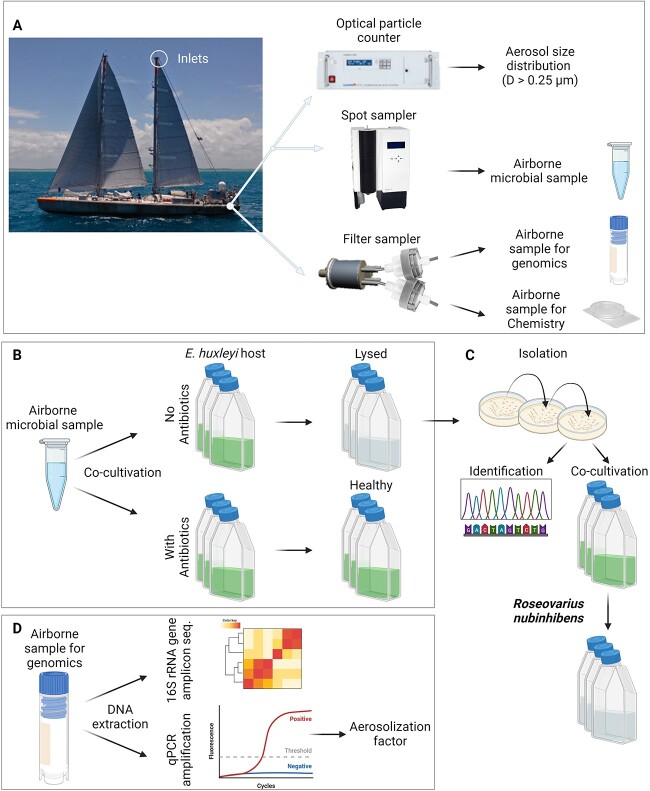
Experimental setup to test the pathogenicity of the collected aerosolized marine bacteria on coccolithophore bloom; marine aerosols were pulled from ~30 m above mean sea level; in one inlet, air samples were split into an OPC, and a Spot-Sampler, collecting air into FSW for periods of ~12 h; from the other inlet, aerosols were collected for periods of ~12 h on PVDF filters for genomic analysis and on polycarbonate filters for SEM-EDS analysis (A); Spot-Sampler’s air samples and controls were cocultivated with healthy *G. huxleyi* cultures, with and without antibiotics (B); demised cultures were used for the isolation and identification of bacterial strains; these bacteria were reintroduced to healthy *G. huxleyi* cultures (C); in parallel, airborne samples and blanks were used for genomic analysis that included both 16S rRNA amplicon sequencing and quantitative PCR for the calculation of the bacterial aerosolization fraction (D); created with BioRender.com.

Measurements were performed aboard the “*R/V* Tara” between May 29 and June 2,2019 [8], around 48.38°N (±0.08) 7.06°W (±0.12). The *R/V* Tara is a 36-m long, 10-m wide aluminium hull schooner with two 27-m long masts (Fig. 1A). It is equipped with a meteorological station (Station Bathos II, Météo France), located on the stern around 7 m above sea level, measuring air temperature and relative humidity (RH). The wind speed and direction are measured at the top of the mast, ~30 m above mean sea level, and a thermo-salinometre (Sea-Bird Electronics SBE45 MicroTSG) measures the sea surface temperature (SST) with its main water entrance located ~0.5–3 m under the sea surface (depending on ocean conditions).

### Sampling strategy

Three aerosol instrumentations were installed on the* R/V* Tara (see Fig. 1A): (i) an optical particle counter (OPC; EDM-180 GRIMM Aerosol Technik Ainring GmbH & Co. KG, Ainring, Germany) for continuous aerosol size distribution measurements (from 0.25 to 32 μm, sorted into 31 bins, every 60 s). A Nafion dryer was installed before the OPC, which reduced the sampled air RH to below 40% [8]. (ii) A Liquid Spot Sampler (Aerosol Devises Inc., Fort Collins, CO, USA) for collecting particles into a small liquid-filled vial containing 1 ml of local Atlantic filtered seawater (FSW) for periods of ~12 h. Blank samples were collected prior to the air sample (except for the first one) by placing the vial with FSW in the instrument, leaving it for a few seconds without turning the pump on (see Tables S1 and S2). After collection, FSW was added to compensate for the evaporated volume (see Table S1). Liquids were then transferred into a 2-ml cryotube using a pipette and sterile tips, and stored at 4°C, to be used for the infection experiments in the lab. The spot sampler was cleaned with 70% ethanol after each sample removal, prior to the attachment of the next sampling tube. (iii) A custom-made aerosol filter system consisting of four 47-mm filter holders and a vacuum pump (Diaphragm pump ME 16 NT, VACUUBRAND BmbH & Co KG, Wertheim, Germany). Three filter holders were loaded with 0.45 μm PVDF membrane filters (HVWP047S6 S-Pak Filters 0.45 μm, 47 mm white plain sterile, Mercury), and one was loaded with a 0.8-μm polycarbonate filter (ATTP04700, Millipore). The filters were replaced twice a day, collecting aerosols for ~12 h (see Table S3), and the filter holders were cleaned with 70% ethanol between filter exchanges. Blank filters were collected every few days by placing filters on the filter holders, closing the system for a few seconds, and reopening the holders (see Table S4). Following sampling, the polycarbonate filters were stored at room temperature in PetriSlide dishes preloaded with absorbent pads (Millipore, PDMA04700) to keep the filters dry while stored, and the PVDF filters were folded into a 2-ml cryotube and plunged into liquid nitrogen. Blank filters were stored under the same conditions as the air samples.

Two separate inlets, located next to each other, one for the OPC and the Spot Sampler and one for the filter system, were constructed out of conductive tubing of 1.9-cm inner diameter and a funnel (allowing the collection of all diameters) and were mounted on the rear backstay of the *R/V *Tara. The inlets were installed at the top of the backstay, ~30 m above mean sea level. The flows through the OPC and the spot sampler were 1.2 l min^−1^ and 1.5 l min^−1^, respectively, and the flow through the filter system was around 30 lpm for each filter.

Seawater samples from the bloom (15 m depth) were collected using an 8-l Niskin bottle as described in Câmara Dos Reis *et al*. (2022) [9].

### Infection experiments and isolation of airborne bacterial strains

In the laboratory, the liquid samples from the Spot-Sampler containing airborne bacteria were cocultivated with *G. huxleyi* strain NCMA374 and NCMA379 (with and without antibiotics; Fig. 1B). These strains were purchased from the National Centre for Marine Algae; Bigelow, ME, USA, originated from the North Atlantic Ocean (NCMA374 from the Gulf of Maine, North America, https://ncma.bigelow.org/CCMP374; NCMA379 from the Eddystone Rocks, English Channel, UK, https://ncma.bigelow.org/CCMP379). The *G. huxleyi* cultures were maintained in FSW (prepared from Coastal Mediterranean seawaters) and a mixture of four different antibiotic types (penicillin, streptomycin, ampicillin, and kanamycin) at 17°C, with a 16-h light/8-h dark illumination cycle (light intensity of 100 mol photons m^−2^ s^−1^ provided by cool white light-emitting diodes). After several dilution cycles of the preserved *G. huxleyi* cultures in FSW + F/2 medium without antibiotics, strains at the exponential growth stage (5–6 × 10^5^ cells/ml) were cocultivated with air samples or with isolates (at 10^5^ bacteria/ml).

The two strains were selected for the initial infection as they previously exhibited viral (NCMA374) [5] and bacterial (NCMA379) [10] susceptibility.

Each culture was exposed in triplicates of 20 ml to 50 𝜇l of the air or blank samples with and without antibiotics (penicillin + streptomycin; Pen-Strep for initial experiments with environmental samples (all Spot Sampler samples were tested, including blank samples), and ampicillin + kanamycin; Amp-Kan for coculturing with isolated bacteria due to indications of resistance to the first antibiotics). Following infection, the culture samples were incubated at 20°C, with illumination cycle as specified above. Each culture was sampled for algal cell and bacterial counts using flow cytometry every 2–3 days.

Infected cultures (with induced demise following coculturing with air samples) were filtered through a 0.8 𝜇m syringe filter and were used to reinfect healthy cultures as described above, as well as to cultivate bacterial populations at 28°C on Marine agar 2216 (MA; Difco) plates for isolation (Fig. 1C). Bacteria were isolated from these plates four times, and the fourth isolated cultures were grown at 28°C in Marine Broth 2216 (MB; Difco) and stored in 15% glycerol at −80°C with duplicates. Each of the isolates was cocultivated with healthy *G. huxleyi* cultures to test the specific algicidal activity of the bacterial strains. The infective isolate was similarly cocultivated with an array of three different axenic *G. huxleyi* strains (detailed in Table S5) to test the range of algicidal activity of the bacterial strain against both naked and calcified strains, as specified above.

To ensure the cultures' axenic nature, a subset of the pre-cultivated *G. huxleyi* cultures was sampled for bacterial cell counts using flow cytometry. Furthermore, postcultivation control cultures (cultivated without bacterial addition) were plated on a Marine agar, with no colonies observed for >10-day incubation period.

### Genomic identification of bacterial isolates

The DNA was extracted from the isolated bacteria using the DNeasy PowerWater Kit (Qiagen, Hilden, Germany), according to the producer’s protocol. The 16S rRNA gene was then amplified using the 16S-27F (AGAGTTTGATCMTGGCTCAG) and 16S-1492R (CGGTTACCTTGTTACGACTT) primers [11] under the following conditions: a PCR mix of 20 μl was prepared using 1X TaqReady mix (Bioline, London, UK), 0.5 μM primers, 2 μl DNA extract, and PCR-grade water (Sigma Aldrich). The thermal conditions included an initial denaturation step at 94°C for 3 min, 35 cycles of denaturation at 94°C for 30 s, annealing at 57°C, for 30 s, and extension at 72°C, for 1.5 min. A final extension step at 72°C was added for 10 min. The PCR products were validated on 1% agarose gel. Amplicons were paired end sequenced using the ABI 3730 DNA Analyzer and were manually assembled using the BioEdit Sequence Alignment Editor software. The assembled and aligned sequences were identified using the BLAST tool from NCBI (see highest identification scores specified in Table S6). The sequences were deposited in the GenBank at NCBI (Project accession numbers: OQ359449-OQ359478).

### Enumeration of algal and bacterial abundances by flow cytometry

On board the *R/V* Tara, *G. huxleyi* cells were analysed immediately after water sampling using the BD Accuri C6 Plus Personal Flow Cytometre (BD Biosciences, USA) by plotting the chlorophyll fluorescence (excitation, 488 nm; emission, 663–737 nm) against the side scatter and were quantified by counting the high-chlorophyll events.

For laboratory infection experiments, *G. huxleyi* and bacterial cells were detected using the CytoFLEX S Flow Cytometre (Beckman Coulter, Nyon, Switzerland). Living algal cell counts were identified by plotting the chlorophyll autofluorescence (excitation at 561 nm, emission at 665–715 nm) versus the forward scatter (FSC) as a proxy for cell size (FSC area threshold = 90 000 arbitrary units (A.U.)). Only cells with an autofluorescence >4.5 × 10^4^ A.U. were enumerated as live cells. Per sample, 200–200 000 events of low- and high-chlorophyll-emitting cells were enumerated. For bacterial counts, samples were fixed with a final concentration of 0.5% glutaraldehyde for at least 30 min at 4°C, then plunged into liquid nitrogen, and stored at −80°C until analysis was carried. After thawing, samples were stained with SYBR Gold (Invitrogen) that was diluted 1:10 000 in tris-EDTA buffer, incubated for 20 min at 80°C, and cooled to room temperature. Bacterial cells were identified by plotting SYBR Gold fluorescence against side scattering (excitation, 488 nm; emission, 500–550 nm; Violet SSC area and FITC area threshold = 1000 A.U.) and were quantified by counting SYBR Gold events (3000–300 000 events were enumerated per sample).

### Extraction of DNA and meta-barcode sequencing

Extraction of DNA from the PVDF air filters was carried out to explore the bacterial community composition and to quantify the specific abundances (Fig. 1D) using the DNeasy PowerWater Kit (Qiagen, Hilden, Germany). Air-sampled filters extraction followed producer’s protocol, for high DNA yield, including heating of the initial reagent, extended incubation times at different extraction steps, repeated final elution step, etc.

The DNA concentrations were evaluated with a Qubit 3.0 Fluorometre (Thermo Fisher, MA, USA), using the DeNovix (Wilmington, DE) dsDNA Ultra High Sensitivity Assay. For DNA sequencing, the bacterial V4–V5 region of the 16S rRNA gene (515F: 5’–GTGYCAGCMGCCGCGGTAA–3′, and 926R: 5’–CCGYCAATTYMTTTRAGTTT–3′) was amplified [12]. A PCR mix of 25 μl was prepared in triplicate using 1× Mytaq mix (Bioline), 0.2 μM primers, 4 μl DNA extract, and PCR-grade water (Sigma Aldrich). A no-template control was included in all runs as well as DNA from a mock community (ZymoBIOMICS Microbial Community DNA Standard; Zymo) [12]. The PCR products were validated on 2% agarose gel, and triplicates were pooled and sent to the DNA Sequencing Facility at the University of Illinois at Chicago. All raw 16S rRNA amplicon sequences were deposited in the Sequence Read Archive (SRA) at NCBI (Project accession numbers: PRJNA901287). DNA sequencing was conducted using Illumina MiSeq sequencing technology (maximum read length of 2 × 300 base pairs).

### Bacterial and algal abundance detection by quantitative PCR

The abundance of *G. huxleyi* Cox3 and *R. nubinhibens* MraZ genes in both water and air samples were determined using quantitative PCR (qPCR, QuantStudio 3 real-time PCR system, Applied Biosystems), using Cox3F1 (5'-AGCTAGAAGCCCTTTGAGGTT-3′ and Cox3R1: 5'-TCCGAAATGATGACGAGTTGT-3′) primers for *G. huxleyi* [13], and self-designed primers for the MraZ gene of *R. nubinhibens* (RN_MraZF1: 5'-AGATACGAAGGGCAGGGTCT-3′ and RN_MraZR1: 5'-GGGAAAGCTCTGACCGTGAA-3'), using the NCBI alignment of the MraZ gene, Primer3 online tool and the NCBI primer BLAST for validation (see Fig. S1). Calibration curves of counted cells ml^−1^ were conducted using DNA extracted from the isolated *R. nubinhibens* and from a *G. huxleyi* NCMA379 culture. Triplicates of 10 μl reaction mixtures consisted of 1× fast SYBR Green master mix (Applied Biosystems), 1 μl extracted DNA, 0.2 μM of each primer, and PCR-grade water (Sigma Aldrich). The thermal cycling conditions consisted of an initial 20 s denaturation and enzyme activation at 95°C, followed by 35 cycles of denaturation at 95°C for 15 s, 15 s annealing at 55°C, and extension at 72°C for 30 s.

### Detection of particulate inorganic carbon

The particulate inorganic carbon (PIC) concentration along the *R/V* Tara’s position was calculated using the Level 3 Aqua-MODIS satellite daily data maps. The daily maps between May 29 and June 2 were averaged to get a composite PIC concentration at each point.

### Particle classification using scanning electron microscopy with energy-disperse X-ray spectroscopy

To classify the elemental composition of the collected particles in the polycarbonate filters, we used a scanning electron microscopy with energy-disperse X-ray spectroscopy (SEM-EDS) and the method as described in Flores *et al*. (2021) [14] together with the particle classification scheme as described in Laskin *et al*. (2012) [15]. We classified each particle with a mean geometrical diameter *D*_geo_ > 0.3 μm into one of five major classes of aerosols: (i) Sea salt: [Na] greater than all other elements detected (except Cl); (ii) Metals with Na: [Na] present but [Na] < [Al, Si, K, Ca, S]; (iii) Sulphate/sea salt: [Na] > [Al, Si, K, Ca] but [Na] < [S]; (iv) Sulphates: [Na] = 0 and [S] > 0; and (v) Other: all remaining particles. The SEM details and EDS spectra acquisition are as described previously [14]. A total of 7857 particles were analysed.

### Atmospheric marine boundary layer and significant height of combined wind waves and swell

The atmospheric marine boundary layer height (AMBLH) and the significant height of combined wind waves and swell (WWSH), in metres, were obtained from the ERA5 reanalysis data [16] provided by the European Centre for Medium-Range Weather Forecasts. The ERA5 AMBLH and WWSH data have a 0.25° spatial and a 1 h temporal resolution. To obtain the ERA5 AMBLH and WWSH data, we calculated the mean within a 1-by-1 degree square (located between 48°and 49° N and 6° and 7° W) during the period when the R/V Tara was above the *G. huxleyi* bloom.

### Statistical analysis

Statistical and community diversity analyses were conducted using R software. The ASV analysis was performed using phyloseq (V. 1.36.0) [17]. A dendrogram was constructed using hierarchical clustering based on the Bray–Curtis dissimilarity index (vegan, V. 2.5-7). The student *t-*test (Excel, V. 2302) was used to compare the differences in the cell counts obtained from flow cytometry between different cocultivation treatments.

## Results

### *G. huxleyi* bloom identification and meteorological conditions

**Figure 2:**
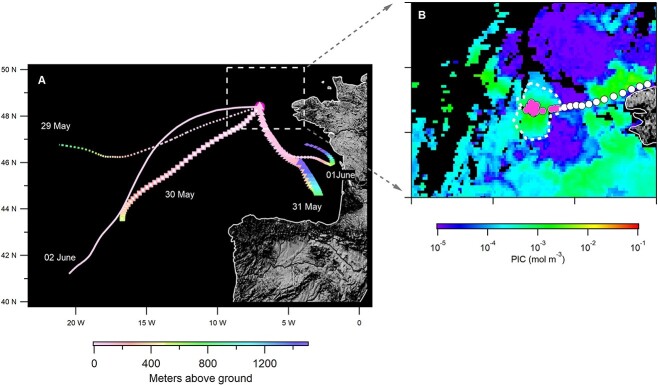
Sampling location and ocean surface PIC concentration. Map showing the sampling location (centred at 48.38°N 7.06°W) with 48-h air mass back trajectories arriving at 50 m height at noon (A); Composite image from MODIS aboard the Aqua satellite between May 29 and June 2, 2019, of particulate inorganic carbon (PIC) levels during sampling. The location of *R/V* Tara is indicated by circles, showing its position within the bloom area (circled with dashed line) during the sampling period and its departure from the bloom on the night of June 2 (B).

**Figure 3:**
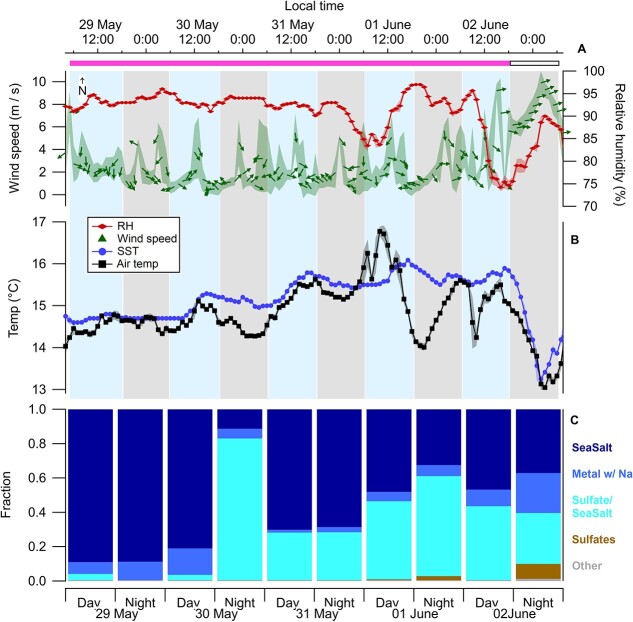
Meteorological conditions during the 5-day sampling period; wind speed measured ~30 m above mean sea level (arrows show the direction and represent the 1-h mean, the shadow represents the standard deviation) and RH measured ~7 m above mean sea level (rhombus) (A); air temperature measured ~7 m above mean sea level (squares) and SST measured between 0.5 and 2 m depth (circles) (B); aerosol composition determined by SEM-EDS for geometrical diameters (*D*_geo_) > 0.3 μm (C); the filled rectangle above the panels correspond to the time duration inside the bloom, as circled in Fig. 2B; the lighter (predominantly day) and darker (predominantly night) bars in panles (A) and (B) represent the measuring period for each filter.

To sample above a coccolithophore bloom, we used satellite data to identify areas of high PIC concentrations in open ocean conditions near Roscoff, France. Once identified, the *R/V* Tara sailed to the approximate centre of the PIC patch and, throughout the sampling period, remained within the same patch of water (Fig. 2). The patch of water was characterized by PIC concentrations of ~1.9 mol m^−3^ (Fig. 2B). Within the bloom, we measured an abundance of calcified *G. huxleyi* cells ranging between 1.6 and 5.6 × 10^3^ cells ml^−1^ in the top 25 m of the ocean surface, confirming we were indeed above a coccolithophore bloom. This cell density can typically be considered as an exponential bloom stage [3, 18]. During the sampling period, wind speeds varied from *U*_30_ = 0.5–9.2 m s^−1^ (Fig. 3A). While a mean wind speed of *U*_30_ = 2.4 (±1.6) m s^−1^ was documented, daily picks of up to ~5.0 m s^−1^ and even higher wind speeds were detected during the last sampling day, with *U*_30_ ~ 4.0 (±2.5) m s^−1^ (Fig. 3A). The air temperature was around 14.9°C (±0.6), while the RH was 91.5% (±4.6). The SST varied around 15.3°C (±0.4) (see Fig. 3A and B). The mean AMBLH and significant height of combined WWSH were ~1243 m (±8) and 3.3 m, respectively (see Fig. S2).

To explore the composition and origin of the sampled marine aerosols, we used back trajectory analysis and the SEM-EDS analysis. The back trajectories (calculated using the NOAA’s HYSPLIT atmospheric transport and dispersion model [19, 20] with a starting height of 50 m), showed the sampled air masses had spent at least 48 h above the ocean before being sampled (Fig. 2A). Additionally, with the obtained approximate chemical signature of the marine aerosols of *D*_geo_ > 0.3 μm with the SEM-EDS (Fig. 3C), we found that over 97% of the particles analysed contained Na, of which 11%–89% were sea salt particles. The back trajectory analysis and the SEM-EDS analysis confirm that the primary type of marine aerosol we sampled originated from the sea surface (*i.e.*, SSAs).

### Marine air incubation with algal cultures

All of the Spot-Sampler’s airborne microbial samples and blanks were initially incubated with *G. huxleyi* strains (NCMA379 and NCMA374, see Table S5). The majority of these samples did not induce a significant decline in algal cells. However, two air samples did induce algal demise. One was collected during May 31, night-time, and the second sample was collected during June 2, daytime (see Table S1). Both exhibited inconsistent responses across replicates. The NCMA374 culture, known to be highly susceptible to viruses showed no effect following inoculation with the air samples. The 0.8-𝜇m filtrates of the demised NCMS379-air sample cultures were reintroduced into healthy cultures with and without antibiotics. This induced a rapid demise, within a few days, in the NCMS379 strain without antibiotics (Fig. S3).

### Bacterial isolation from air sample-infected cultures

We isolated 30 bacterial strains from the air-infected NCMA379 cultures (Samples 6 and 9 in Table S1) to identify potential bacterial pathogens that can lead to cell lysis in the algal host. Sanger sequencing of the 16S rRNA gene from the isolated bacteria revealed a total of five different taxa (see Table S6): 24 isolates were identified as *Pseudoaltermonas* sp*.* (99.1% identification similarity with six different *Pseudoaltermonas* species), 4 as *R. nubinhibens* (99.0% identification similarity), 1 as *Idiomarina* sp*.* (99. 6% identification similarity), and 1 as *Marinobacter koreensis* (98.2% identification similarity)*.* One additional strain was identified as *Micrococcus* sp., with <75% identification similarity (see Table S6), concluded to be a handling contaminant.

**Figure 4:**
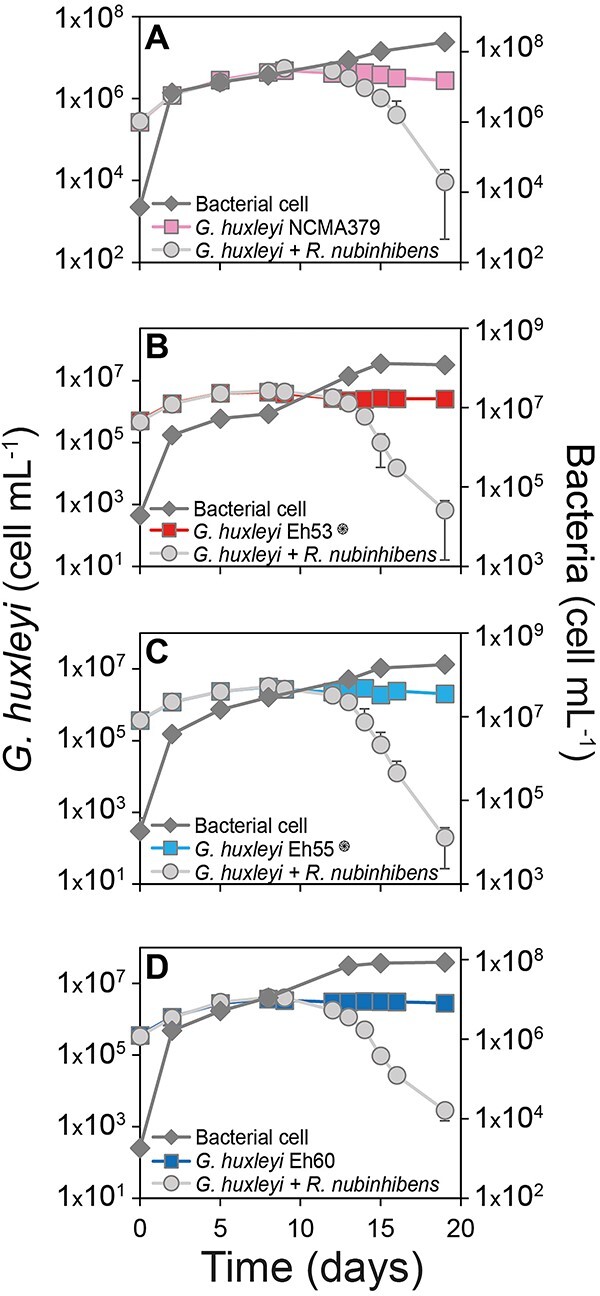
*Roseovarius nubinhibens* isolated from air samples kills various natural *G. huxleyi* strains in the lab; detailed time courses of *G. huxleyi* cells (A—NCMA379; B—Eh53; C—Eh55; D—Eh60, more information is given in Table S5) incubated with (circles) and without (squares, as a control) air sample-isolated *R. nubinhibens* and the bacterial cell growth in the monocultures ( rhombus), represented by the difference between the infected and the control cultures; the symbol 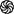 denotes calcified strains.

Each of the isolates was inoculated into healthy *G. huxleyi* cultures to examine the pattern of algal response to the presence of bacteria (Fig. S4). Only *R. nubinhibens* showed a robust pathogenicity against NCMA379, with a significant reduction in algal cell counts compared to the control (Fig. 4A; Two sample *t*-tests assuming unequal variances against blank sample, Exp. day = 18, *P* < .0001). The bacterial growth in the infected culture was also significantly higher compared to the control, increasing in four orders of magnitude in coculture with the algal host cells (two sample *t*-tests, unequal variances against blank sample, Exp. day = 18, *P* = .004). The growth dynamics of the alga-bacterium exhibited a typical bi-phasic response in which, at the first 13 days, they showed a mutualistic interaction and a second pathogenic phase that led to algal decline (Fig. S5A). No algal mortality was observed for the NCMA374 (Fig. S6), suggesting a strain-specificity in the virulence of *R. nubinhibens*. Inoculation of *G. huxleyi* with different initial *R. nubinhibens* cell concentration showed algal demise of all treatments, while bacterial cell densities increased accordingly, with faster algicidal effect at higher bacterial densities. This demonstrates the dependence of algal cell death on specific threshold of bacterial densities during coculture (Fig. S5A and B).

We additionally screened for the pathogenicity host range of *R. nubinhibens* against other three *G. huxleyi* cultures, both naked and calcified strains, isolated from *G. huxleyi* induced bloom in a mesocosm in Bergen 2018 [21] (see Table S5). The *R. nubinhibens* demonstrated pathogenicity against all three *G. huxleyi* strains, and it is presented in Fig. 4B–D. The capability of *R. nubinhibens* to infect an array of naturally isolated strains with varying morphologies (both naked and calcified), signifies the ecological significance of this bacterial pathogen in the natural bloom demise.

### Detection of the isolated bacteria in airborne microbiome

To verify the presence of the isolated bacteria in the aerosolized microbial composition, we analysed the DNA extracted from air filters collected onboard the *R/V* Tara. We further conducted a screening for air-isolated marine bacteria in the 16S rRNA gene identified sequences (Fig. S7) and observed that the air-isolated marine bacteria were detected in the microbiome of air filter samples. These bacteria were absent from the *G. huxleyi* cultures microbiome, reaffirming they did not originate from the cultures. Instead, our results show that these bacteria originated from the sampled air above the natural bloom.

### Estimating *R. nubinhibens* emission from the ocean to the atmosphere

**Figure 5:**
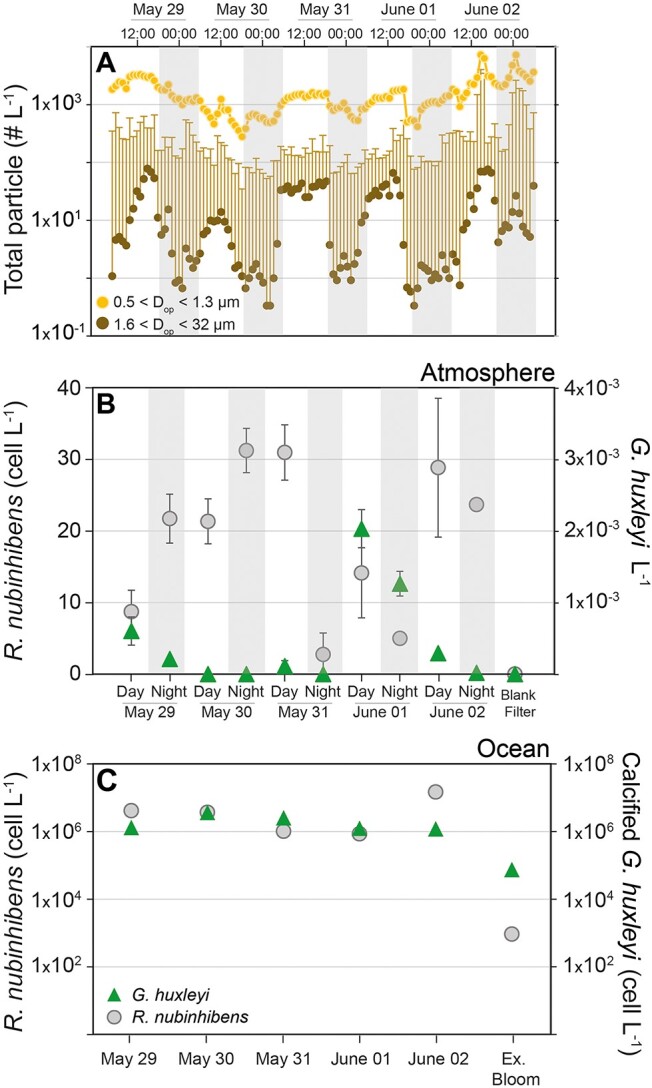
Abundance of marine aerosols, *R. nubinhibens*, and *G. huxleyi* in the AMBL**;** The concentration of marine aerosols above the bloom for 0.5 < *D*_op_ < 1.3 μm (orange), and 1.6 < *D*_op_ < 32 μm (brown) (A), as well as the abundance of the MraZ gene of *R. nubinhibens* (circles) and the *Cox3* gene (triangles) from *G. huxleyi* in air filter samples (B), and the MraZ gene of *R. nubinhibens* and the calcified *G. huxleyi* cell counts per litre of the water samples collected at ~15 m depth (C); results in (B) and (C) represent the average of three replicates (*n* = 3), and the error bars are 1σ; the shaded bars represent predominantly nighttime samples, and signify the measuring period for each filter; Ex. Bloom refers to the area outside of the *G. huxleyi* bloom.

To estimate the approximate abundance of the airborne *R. nubinhibens* in the sampled air over the bloom, we first evaluated the total marine aerosol abundance using the online OPC for particles in diameter between 0.5 and 1.3 μm (*N*_0.5–1.3_; Fig. 5A). The abundance of *R. nubinhibens* was then evaluated in the DNA extracted from the air filter and water samples, as estimated by qPCR using primers targeting the cell division protein MraZ gene (Fig. 5B). We found an average *N*_0.5–1.3_ = 1.584 $\times$ 10^3^(±0.797$\times$ 10^3^) total particle counts l^−1^ air (Fig. 5A) and an average of 20.8 (± 9.5) *R. nubinhibens* cell-equivalent l^−1^ air (at the same order of magnitude as other reported airborne rates [22, 23]; Fig. 5B). If *R. nubinhibens* is unattached to larger airborne particles, this suggests that ~1.3% of aerosols with diameters between 0.5 and 1.3 μm might represent the upper limit of *R. nubinhibens* abundance in the atmosphere above the algal bloom. While a dramatic decrease in the total particle concentration of *D* > 1.3 μm has been detected (an average *N*_01.6–32_ = 16.3 (±24) counts l^−1^ air; Fig. 5A), it cannot be ruled out that the transfer of these bacteria may occur by attachment to larger particulates (e.g., algal fragments).

The aerosolization factors (defined as the relative enrichment of a selected microbial group between the sea and air) of *R. nubinhibens* and *G. huxleyi* were evaluated by calculating the ratio of the airborne abundance to that found in the water, as previously described by Michaud *et al*. (2018) [24]. The *G. huxleyi* Cox3 gene showed four orders of magnitude lower aerosolization factor (3.05 × 10^−10^ ± 2.08 × 10^−10^) compared to 4.26·10^−6^ ± 2.65·10^−6^ of the *R. nubinhibens* MraZ gene. The later bacterial ratio is similar to that of lab-tested viral infection of *G. huxleyi* culture [5]. The difference between the emission rates of *G. huxleyi* and *R. nubinhibens* cells may indicate an independent emission of the pathogenic bacterium with no association to the emitted *G. huxleyi* cell fragments. The alternative emission of ~10 000 bacteria per 1 *G. huxleyi* cell is less likely. Independent bacterial emission would indicate a smaller size fraction of these bioaerosols, which are expected to have a longer atmospheric lifetime of hours or even days, thus, larger dispersal distance.

## Discussion

Bacterial pathogenicity towards algal host cells have been documented in several algal species [25, 26]. Recent studies have revealed “Jekyll-and-Hyde” dynamics in several algae–bacteria interactions in which a shift in the bacterial lifestyle from mutualism to pathogenicity depends on the algal physiological and metabolic states [10, 27–29]. Several bacterial pathogens that possess algicidal effect towards *G. huxleyi* host cells were isolated, including *Sulfitobacter* D7 sp., *Phaeobacter inhibens*, and *Pseudoalteromonas piscicida*. To date, the metabolic crosstalk and chemical signalling between algae and bacteria have been mainly characterized in laboratory cultures. Thus, the ecological significance of these important interactions in the context of natural algal blooms is still requires further investigations.

Here, we report the isolation of a new bacterial pathogen from air samples collected above *G. huxleyi* bloom in the North Atlantic. This bacterium, *R. nubinhibens*, from the *Roseobacter* clade (the Rhodobacteraceae family), exhibited an algicidal activity towards several strains of *G. huxleyi*, including isolates from algal blooms. The *R. nubinhibens* is recognized for its ability to cleave dimethylsulfoniopropionate and to generate the climate relevant volatile, dimethyl sulphide, or, alternatively, demethylate into methanethiol [30]. Akin *R. nubinhibens*, several other bacterial pathogens as *Sulfitobacter* D7 sp. and *P. inhibens* belong to the Rhodobacteraceae family, which is typically associated with *G. huxleyi* blooms. [10, 27, 31, 32].

While previous laboratory experiments have showed that marine aerosolized viruses can infect *G. huxleyi* culture [5], our study provides novel evidence of the presence of the bacterium *R. nubinhibens* in the atmospheric marine environment above a natural *G. huxleyi* bloom. Under most atmospheric conditions, the marine boundary layer for particle concentrations with *D*_dry_ < 10 μm is known to be well mixed [33], implying a vertical distribution of such marine bioaerosols throughout the atmospheric marine boundary layer (AMBL). However, the microbial composition might not be uniform across the AMBLH, as the airborne microbial composition will be sorted by the lifetime of the particles it is attached to in the atmosphere. The difference in suspension time, varying between minutes for large aerosols and several days for smaller ones, influences the microbial vertical spread as well as the distance it can be carried in the atmosphere away from its source. Consequently, the observed composition will likely be different in the higher levels of the AMBL where larger aerosols are less likely to be abundant. However, given the well-mixed nature of the AMBL, the presence of *R. nubinhibens* is most likely throughout the AMBLH. Our findings suggest that *R. nubinhibens* can remain infectious after being emitted into the atmosphere and may induce pathogenicity against a specific bloom-forming algal host.

Our results further indicate an emission ratio of 1:10^6^ pathogenic cells to the air over an *G. huxleyi* bloom. Assuming they remain viable for several hours, these bacteria could travel tenths of kilometres to infect other bloom patches. As a typical *G. huxleyi* bloom may last in average for several weeks [3], the aerial transport could be an additional important mechanism of infection spreading.

Recently, there have been documented isolations of pathogenic bacteria that can infect other phytoplankton groups such as diatoms [34, 35]. Therefore, the aerial infection mechanism of bacterial pathogens may have a broad implication to diverse algal–bacteria interactions.

To the best of our knowledge, the observed demise of *G. huxleyi* strains following coculturing with an airborne pathogenic bacterium has not been reported before. This indicates its potential pathogenicity against a natural *G. huxleyi* bloom and suggests that airborne marine bacteria may have an unappreciable role in the open ocean’s large-scale infection of algal blooms.

## Supplementary Material

SI_Lang-Yona_18-01-24_wrae016

## Data Availability

The data generated or analysed during this study are included in this published article and its Supplementary Information files. The raw 16S rRNA amplicon sequences were deposited in the SRA at NCBI (Project accession numbers: PRJNA901287). The assembled and aligned sequences of the isolated airborne bacteria were deposited in the GenBank at NCBI (Project accession numbers: OQ359449-OQ359478).
