# Supplementary material for: Impact of airborne algicidal bacteria on marine phytoplankton blooms
Source: ISME J. 2024 Mar 5;18(1):wrae016. doi: 10.1093/ismejo/wrae016 (PMC10944695; doi:10.1093/ismejo/wrae016)
Supplement: SI_Lang-Yona_18-01-24_wrae016 [file si_lang-yona_18-01-24_wrae016.pdf]

Supplementary materials for

Impact of airborne algicidal bacteria on marine phytoplankton blooms.

Authors:

Naama Lang-Yona[1§*], J. Michel Flores[2*], Tal Sharon Nir-Zadock[1], Inbal Nussbaum[1], Ilan Koren[2], Assaf Vardi[1]

Affiliations:

[1]Weizmann Institute of Science, Department of Plant and Environmental Science, 7610001 Rehovot, Israel.

[2]Weizmann Institute of Science, Department of Earth and Planetary Sciences, 7610001 Rehovot, Israel.

[§]Current affiliation: Civil and Environmental Engineering, Technion - Israel Institute of Technology, 3200003 Haifa, Israel.

*These authors have contributed equally to this work

Corresponding authors:

Assaf Vardi, assaf.vardi@weizmann.ac.il, Ilan Koren, Ilan.Koren@weizmann.ac.il.

Table of Content

Table of Content 2

Supplementary Figures 3

Figure S1 3

Figure S2 4

Figure S3 5

Figure S4 6

Figure S5 7

Figure S6 7

Figure S7 9

Supplementary Tables 10

Table S1: Spot sampler samples. 10

Table S2: Spot sampler Blanks. 10

Table S3: Air filter samples. 11

Table S4: Blank filter samples. 11

Table S5: Isolate strains co-cultivated with air-isolated *Roseovarious nubinhibens*. 12

Table S6: Identification score of representative isolates. 13

Supplementary Figures

Figure S1.

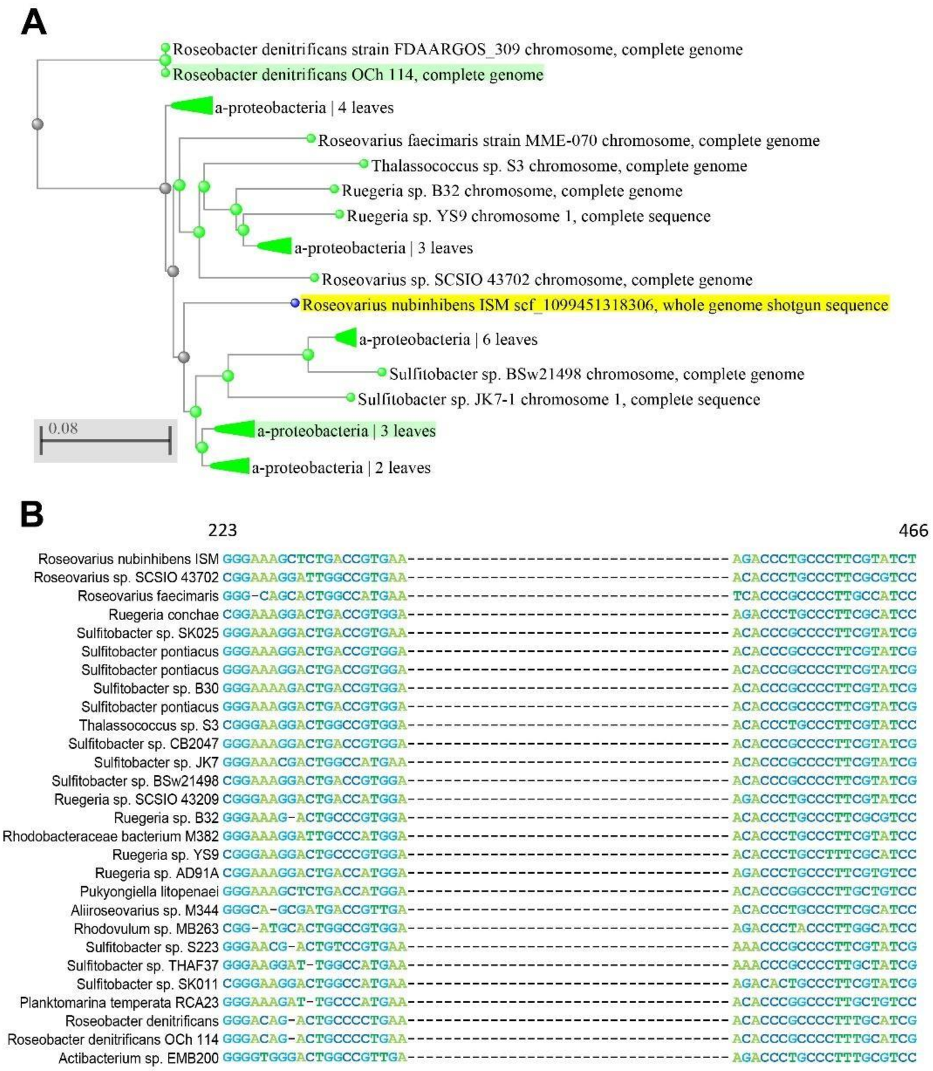

Figure S1: **Genomic Presence of MraZ Gene and Primer Specificity Assessment.** The MraZ gene is found in 28 genomes in the NCBI database, of which, 15 have less than 0.075 distance compared to the *R. nubinhibens* genome (**A**). The alignment of the designed primers against all strains shows only *R. nubinhibens* genome had the primer sequences (**B**).

Figure S2.

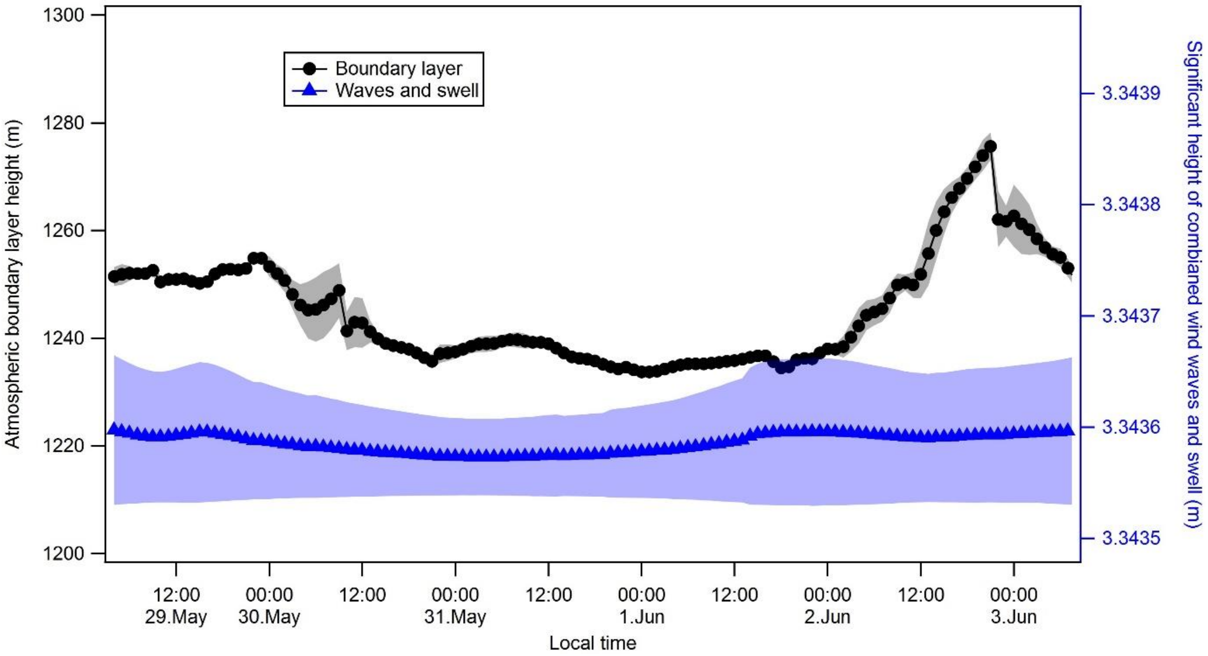

Figure S2: **Ocean-Atmosphere height dynamics.** The calculated Atmospheric boundary layer (Black circles) and the significant height of combined wind waves and swell (blue triangles) are denoted in meters.

Figure S3.

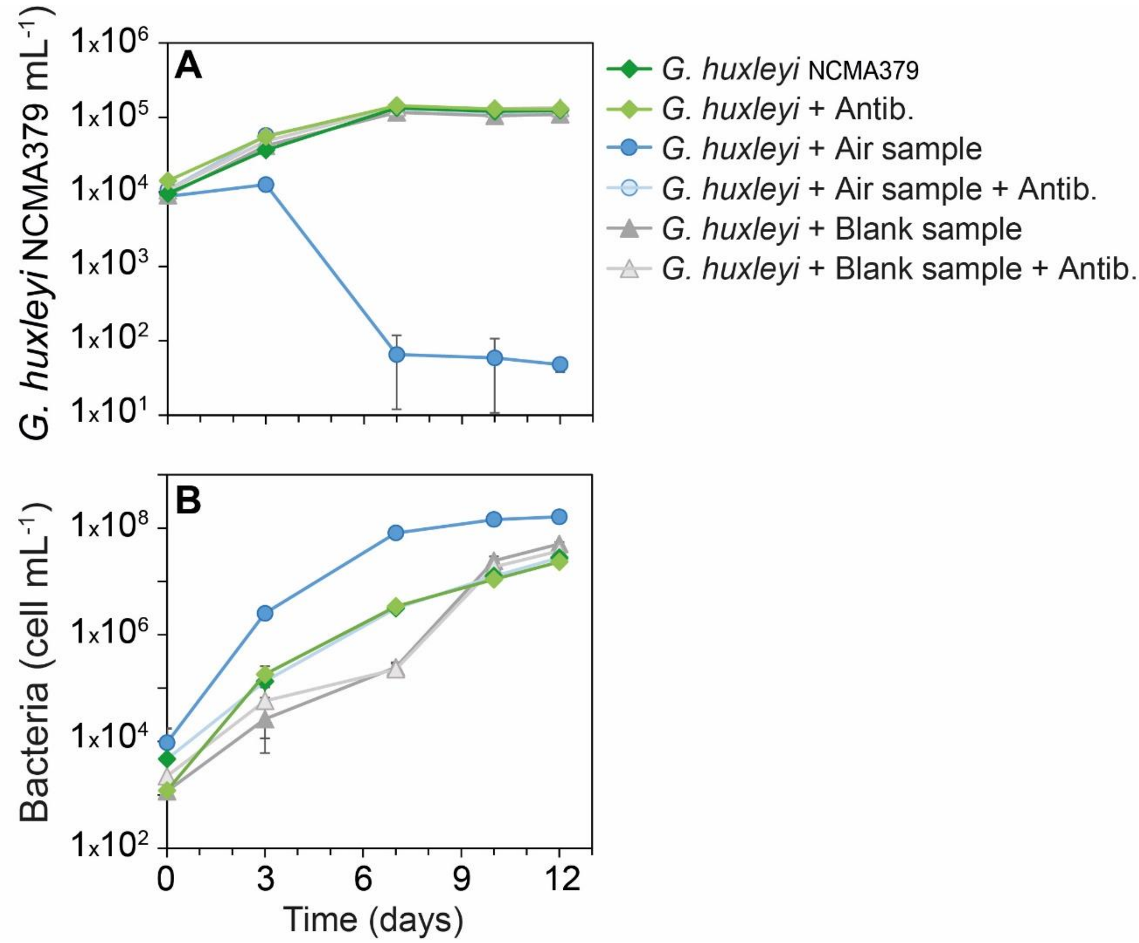

Figure S3: Co-culturing of *G. huxleyi* NCMA379 strain with air samples. Detailed time courses of *G. huxleyi* NCMA379 following co-culturing with and without antibiotics (penicillin+streptomycin) with filtered co-cultured air (circle), and control (triangle) samples, as well as the monoculture alone (green rhombus) (**A**), bacterial growth during co-culturing (**B**). Results represent the average of three replicates (n = 3), the error bars are 1σ. Two sample *t*-test assuming unequal variances was tested against blank sample for A (p = 0.0078) and B (p = 0.033).

Figure S4.

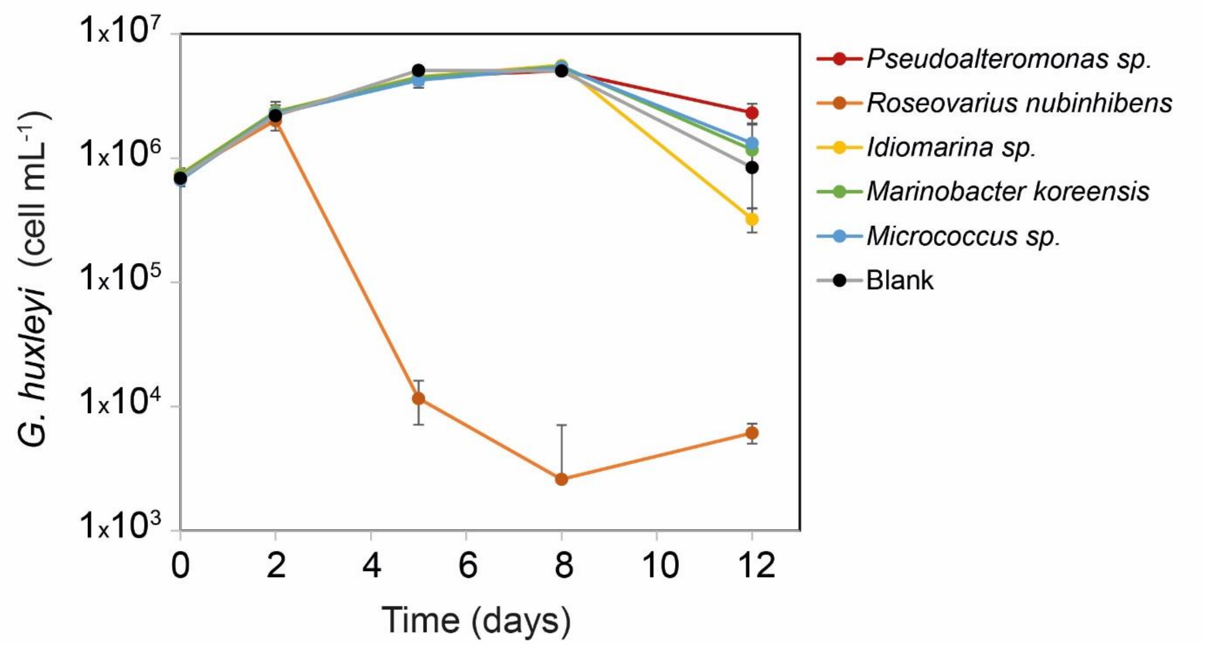

Figure S4: **Isolate infection of *G. huxleyi* NCMS379.** A detailed time course of *G. huxleyi* NCMA379 cell growth, following co-cultivation with the different airborne isolates. Results represent the average of three replicates (n = 3), the error bars are 1σ. To confirm infectivity, all bacteria were uniformly added at a concentration of 10^7 cells ml^{-1} in this specific experiment. This probably induced an infection at an earlier stage, seen by the reduction in cell counts in this particular test compared to the other incubation experiments.

Figure S5.

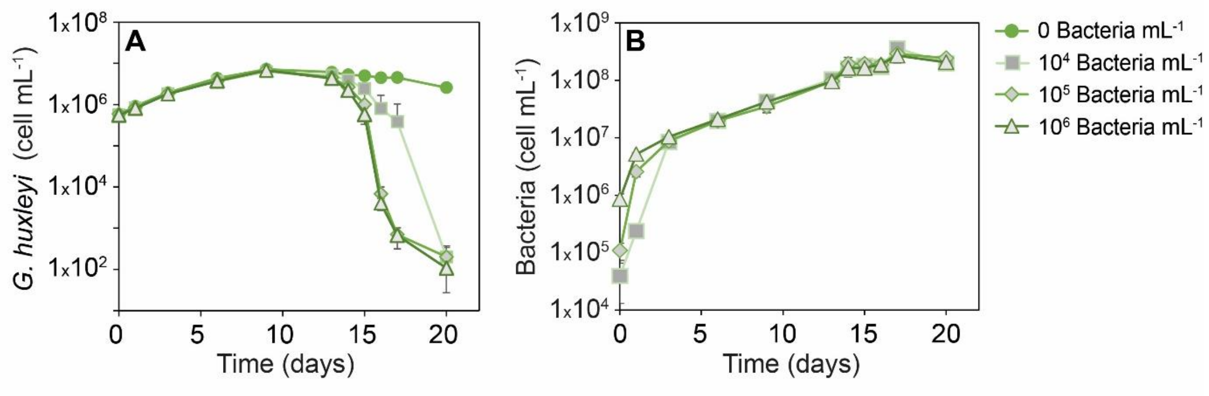

Figure S5: **Temporal dynamics of co-incubated *G. huxleyi* NCMA379 and *Roseovarius nubinhibens* growth patterns.** A detailed time course of *G. huxleyi* NCMA379 (**A**) as well as *G. huxleyi* NCMA374 (**B**) with *R. nubinheibens* following co-incubation, demonstrating their inverse nature of growth starting from the late growth phase, and the difference in *G. huxleyi* NCMA379 cell growth (**C**) and bacterial growth (**D**), for different initial bacterial cell concentrations. Results represent the average of three replicates (n = 3), the error bars are 1σ.

Figure S6.

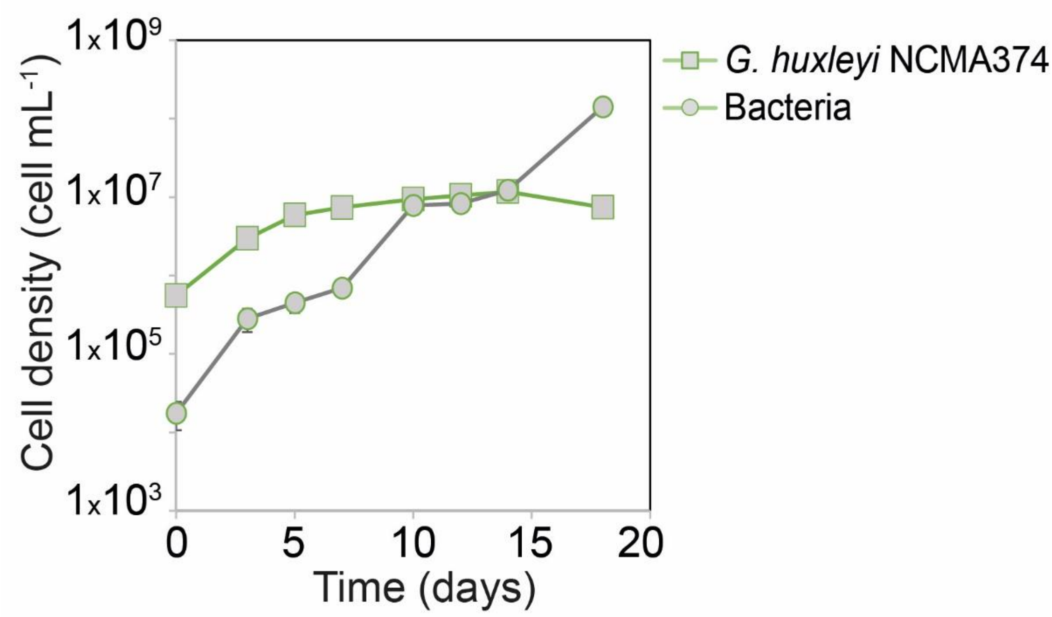

Figure S6: **Temporal dynamics of co-incubated *G. huxleyi* NCMA374 and *Roseovarius nubinhibens* growth patterns.** A detailed time course of *G. huxleyi* NCMA379 cell (square) and bacterial (circles) growth, demonstrating coexistence and resilience of the algal culture to *R. nubinhibens*. Results represent the average of three replicates (n = 3), the error bars are 1σ.

Figure S7.

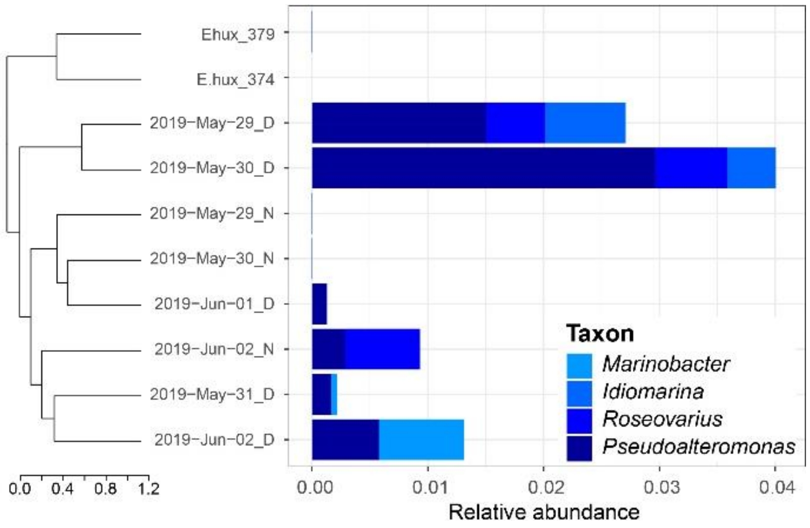

Figure S7: **Isolated Bacterial genera found in DNA extracted from air filters.** The relative abundance of the closest match of identification to the bacterial air isolates based on 16S rRNA gene amplicon sequencing following the exclusion of blank-detected ASVs. The samples are clustered based on microbial composition similarity.

Supplementary Tables

Table S1: Spot sampler samples. The initial, final, and total spot-sampling time in hours, the evaporated volume compensated with filtered sea water in µl post sampling, the particle > 0.5 µm average counts per cm^{-3}, and total concentrations evaluated from the online OPC analysis.

Sample #	Collection times: Initial (DD-MM-YY hh:mm)	Final (DD-MM-YY hh:mm)	Total (h)	Added FSW post sampling (µl)	Avg conc (# cm^{-3}) D > 0.5µm	Total particles D > 0.5µm
1	29-05-19 11:31	29-05-19 18:28	6.95	300	3.17(±0.5)	1548590
2	29-05-19 18:32	30-05-19 05:35	11.05		1.71(±0.57)	1137900
3	30-05-19 05:40	30-05-19 18:10	12.5	300	0.93(±0.38)	696217
4	30-05-19 18:23	31-05-19 06:05	11.7		0.60(±0.17)	423047
5	31-05-19 06:28	31-05-19 18:27	11.98	500	1.70(±0.24)	1221120
6	31-05-19 18:30	01-06-19 06:07	11.62		1.03(±0.41)	716099
7	01-06-19 06:20	01-06-19 18:22	12.03	400	1.66(±0.46)	1191750
8	01-06-19 18:36	02-06-19 06:17	11.68		1.02(±0.38)	713988
9	02-06-19 06:34	02-06-19 18:05	11.52		3.25(±2.87)	2245700
10	02-06-19 18:16	03-06-19 06:19	12.05	1000	3.74(±2.08)	2706890

Table S2: Spot sampler Blanks. The date and time of the blank filters are specified.

**Collection Date** (DD-MM-YY)	**Collection time** (hh:mm)
29-05-19	18:29
30-05-19	05:36
30-05-19	18:15
31-05-19	06:15
31-05-19	18:28
01-06-19	06:10
01-06-19	18:25
02-06-19	06:20
02-06-19	18:10

Table S3: Air filter samples. The initial, final, and total filter-sampling time in hours, the particle > 0.5µm average counts per cm^{-3} and total concentrations evaluated from the online OPC analysis.

Sample #	Collection times			Avg conc (# cm^{-3}) D > 0.5µm	Total particles D > 0.5µm	Wind speed (m /s)
	Initial (DD-MM-YY hh:mm)	Final (DD-MM-YY hh:mm)	Total (h)			
1	29-05-19 05:02	29-05-19 18:05	13.05	2.99(±0.97)	2338390	3.01 (±1.80)
2	29-05-19 18:20	30-05-19 05:40	11.33	1.73(±0.59)	1174890	2.37 (±1.70)
3	30-05-19 05:50	30-05-19 18:15	12.42	0.92(±0.37)	684307	1.46(±1.65)
4	30-05-19 18:22	31-05-19 06:09	11.78	0.61(±0.18)	427788	1.75(±1.96)
5	31-05-19 06:21	31-05-19 18:19	11.97	1.70(±0.25)	1216020	2.23 (±2.05)
6	31-05-19 18:30	01-06-19 06:12	11.7	1.03(±0.41)	721316	1.73(±1.63)
7	01-06-19 06:20	01-06-19 18:28	12.13	1.65(±0.46)	1195130	2.29(±1.96)
8	01-06-19 18:36	02-06-19 06:22	11.77	1.02(±0.39)	722444	2.35(±2.05)
9	02-06-19 06:34	02-06-19 18:10	11.6	3.25(±2.86)	2264380	3.80(±3.04)
10	02-06-19 18:16	03-06-19 06:19	12.05	3.74(±2.08)	2706890	7.58(±1.50)

Table S4: Blank filter samples. The date and time of the blank filters are specified.

**Collection Date** (DD-MM-YY)	**Collection time** (hh:mm)
29-05-19	18:08
31-05-19	06:12
02-06-19	06:25

Table S5: Isolate strains co-cultivated with air-isolated *Roseovarious nubinhibens*. The strain name, catalogue number, culture collection, origin of the isolated culture and the calcification state are listed for each of the tested cultures.

Strain name	Cat. number	Culture collection	Origin	Calcified
CCMP379	Plymouth 92	NCMA, Bigelow, ME, USA	English Channel, Europe	-
CCMP374	Plymouth 92	NCMA, Bigelow, ME, USA	English Channel, Europe	-
Bergen-EH53	RCC6960	RCC, Roscoff, France	Bergen fjord, Norway	v
Bergen-EH55	RCC6962	RCC, Roscoff, France	Bergen fjord, Norway	v
Bergen-EH60	RCC6967	RCC, Roscoff, France	Bergen fjord, Norway	-

Table S6: Identification score of representative isolates. The scientific name, Maximal score of identification, query coverage, degree of identity (%), the length of accession sequence (Acc. Len), and the accession value.

Scientific Name	Max Score	Query Cover	Identity (%)	Acc. Len	Accession
**Isolated strain 1**					
Roseovarius nubinhibens	2416	96%	98.96	1418	NR_028728.1
Roseovarius sp. JB-20	2407	96%	98.89	1355	KP265960.1
uncultured *Roseobacter* sp.	2399	96%	98.74	1368	JX530607.1
Roseovarius nubinhibens	2399	96%	98.74	1412	MW965564.1
Roseovarius nubinhibens	2390	96%	98.95	1335	MK045795.1
Roseovarius nubinhibens	2383	95%	99.02	1325	LT600603.1
Roseovarius nubinhibens	2383	95%	99.02	1321	MW629879.1
Roseovarius nubinhibens	2383	95%	98.95	1323	MN749916.1
Roseovarius nubinhibens	2381	96%	98.66	1341	MW675175.1
Roseovarius nubinhibens	2375	95%	99.02	1320	LT600600.1
Roseovarius nubinhibens	2372	95%	99.02	1317	LT600604.1
Roseovarius nubinhibens	2370	95%	98.65	1324	MN749904.1
Roseovarius nubinhibens	2368	95%	98.94	1318	LT600535.1
**Isolated strain 2**					
Pseudoalteromonas agarivorans	2442	92%	99.12	4E+06	CP033065.1
Pseudoalteromonas sp. YZZY-F7	2442	92%	99.12	1397	KX001927.1
Pseudoalteromonas atlantica	2442	92%	99.12	1371	OQ071787.1
Pseudoalteromonas sp. AB293f	2442	92%	99.12	1477	FR821202.1
Pseudoalteromonas sp. NBRC 101683	2442	92%	99.12	1461	AB681527.1
Pseudoalteromonas espejiana	2440	92%	99.12	1411	OQ216737.1

Scientific Name	Max Score	Query Cover	Identity (%)	Acc. Len	Accession
**Isolated strain 3**					
Idiomarina sp.	2479	92%	99.56	1402	MN031333.1
Idiomarina sp.	2479	92%	99.56	1383	MN031316.1
Idiomarina abyssalis	2479	92%	99.56	1377	KM407668.1
Idiomarina sp. WM21	2479	92%	99.56	1476	HG004197.1
Idiomarina abyssalis	2479	92%	99.56	1410	ON754250.1
Idiomarina abyssalis	2479	92%	99.56	3E+06	CP081832.1
Idiomarina sp.	2479	92%	99.56	1388	KC753341.1
Idiomarina sp.	2479	92%	99.56	1446	MT921728.1
Idiomarina sp. FG-5	2479	92%	99.56	1418	JF521499.1
Idiomarina sp. H96B18	2479	92%	99.56	1451	FJ746576.1
Idiomarina sp. SB24	2479	92%	99.56	1439	EU308439.1
Idiomarina sp. NT N144	2479	92%	99.56	1466	AB167046.1
Idiomarina sp. SMT L2	2479	92%	99.56	1465	AB166939.1
**Isolated strain 4**					
Marinobacter koreensis	2507	97%	98.25	1481	DQ097526.1
Marinobacter salarius	2495	97%	98.11	4E+06	CP020931.1
Marinobacter sp.	2495	97%	98.11	1462	OL630560.1
Marinobacter sp.	2495	97%	98.11	1462	OL630556.1
Marinobacter algicola	2490	97%	98.04	1498	MK493536.1
Marinobacter sp.	2490	97%	98.04	1516	MN099579.1
Marinobacter salarius	2490	97%	98.04	1458	CP021333.1
Marinobacter sp.	2490	97%	98.04	1458	OP159063.1
Marinobacter sp. NP-1383C-30R	2490	97%	98.04	1462	KJ914666.1
Marinobacter sp. SCS75m-2	2490	97%	98.04	1451	JX533669.1
Marinobacter salarius	2490	97%	98.04	1432	MT645874.1
Marinobacter salarius	2490	97%	98.04	1461	MT645873.1

Scientific Name	Max Score	Query Cover	Identity (%)	Acc. Len	Accession
**Isolated strain 5**					
Micrococcus yunnanesis	625	72%	75.45	1426	NR_116578.1
Micrococcus luteus	619	72%	75.33	1525	NR_075062.2
Micrococcus luteus	619	72%	75.33	1418	NR_037113.1
Micrococcus luteus	619	72%	75.33	1325	NR_114673.1
Micrococcus aloeverae	614	72%	75.21	1411	NR_134088.1
Micrococcus endophyticus	614	72%	75.21	1438	NR_044365.1
